# Long-term adverse neuropsychological functioning in children who survived meningococcal septic shock: is there a relationship with sedation and analgesia during paediatric ICU admission?

**DOI:** 10.1186/cc10942

**Published:** 2012-03-20

**Authors:** HL Van Zellem, E Utens, SN De Wildt, WC Hop, NJ Vet, KF Joosten, C Buysse

**Affiliations:** 1 Erasmus MC - Sophia Children's Hospital, Rotterdam, the Netherlands; 2 Erasmus MC, Rotterdam, the Netherlands

## Introduction

Our objective was to evaluate the association between the use of sedative and analgesic agents during paediatric intensive care unit (PICU) treatment and long-term neuropsychological outcome in children who survived meningococcal septic shock (MSS).

## Methods

This study is part of a medical and psychological follow-up study of all consecutive MSS survivors requiring PICU treatment between 1988 and 2001 at the Erasmus MC - Sophia Children's Hospital, a tertiary-care university hospital. This follow-up study revealed that MSS survivors showed long-term (at least 4 years after PICU admission) impairments on several domains of neuropsychological functioning. Severity of illness was no significant predictor of adverse neuropsychological outcome. The use (type, number and dose) of sedatives and analgesics was retrospectively evaluated.

## Results

The study population consisted of 77 patients (52% male (*n *= 40), median age 25 months at time of PICU admission). In 45 patients (58%) one or more analgesic and/or sedative drugs were administered during PICU admission. Benzodiazepines were the most commonly used drugs (*n *= 39; 51%), followed by opioids (*n *= 23; 30%). In total 15 different kinds of analgesic or sedative drugs were given. There was a statistically significant correlation between the use of opioids (both as continuous (cumulative dose) and dichotomous variable) and adverse outcome on multiple domains of neuropsychological functioning (full-scale IQ (*P *= 0.02; *Z *= -2.28), verbal IQ (*P *= 0.02; *Z *= -2.32), verbal reasoning (*P *= 0.02; *Z *= -2.34), social comprehension (*P *= 0.01; *Z *= -2.56), visual-motor integration (*P *= 0.03; *Z *= -2.17)). After univariate analysis, correcting for socioeconomic status, age at follow-up and severity of illness, these correlations remained significant.

## Conclusion

The use of opioids during PICU admission was significantly associated with long-term adverse neuropsychological outcome in MSS survivors.

